# Focus on the Interactive Cooperation Among Mechanotransduction and Biochemical Processes in Pancreatic Ductal Adenocarcinoma Development and Possible Adjuvant Role of Retinoic Acid for Its Treatment: A Narrative Review

**DOI:** 10.3390/cancers18121932

**Published:** 2026-06-13

**Authors:** Sirio Fiorino, Wandong Hong, Dario de Biase, Laura Mastrangelo, Francesca Maccioni, Alfonso Grottesi, Francesca Ambrosi, Luca Pincigher, Federico Lari, Christian Bergamini, Elio Jovine, Maddalena Zippi

**Affiliations:** 1 Internal Medicine Unit, Budrio Hospital, Budrio (Bologna), Azienda USL, 40065 Bologna, Italy; f.lari@ausl.imola.bo.it; 2 Department of Gastroenterology and Hepatology, The First Affiliated Hospital of Wenzhou Medical University, Wenzhou 325015, China; xhnk-hwd@163.com; 3 Department of Pharmacy and Biotechnology (FaBit), University of Bologna, 40126 Bologna, Italy; dario.debiase@unibo.it (D.d.B.); luca.pincigher2@unibo.it (L.P.); christian.bergamini2@unibo.it (C.B.); 4 Solid Tumor Molecular Pathology Laboratory, IRCCS Azienda Ospedaliero-Universitaria di Bologna, 40138 Bologna, Italy; 5 Department of Surgery, AOU Sant’Orsola Malpighi, IRCCS Azienda Ospedaliera Universitaria, 40138 Bologna, Italy; laura.mastrangelo@ausl.bologna.it (L.M.); elio.jovine@ausl.bologna.it (E.J.); 6 Department of Radiological, Oncological and Pathological Sciences, Umberto I Hospital, Sapienza University of Rome, 00161 Rome, Italy; francesca.maccioni@uniroma1.it; 7 Unit of General Surgery, Sandro Pertini Hospital, 00157 Rome, Italy; alfonso.grottesi@aslroma2.it; 8 Pathology Unit, DIAP—Dipartimento Interaziendale Anatomia Patologica, Maggiore Hospital-AUSL Bologna, 40133 Bologna, Italy; francesca.ambrosi@ausl.bologna.it; 9 Unit of Gastroenterology and Digestive Endoscopy, Sandro Pertini Hospital, 00157 Rome, Italy; maddyzip@yahoo.it

**Keywords:** pancreatic cancer, KRAS, p53, PUMA, PRMT5, NRF2, ROS, oxygen tension, oxidative stress, retinoic acid, redox therapy

## Abstract

**Background**: Pancreatic ductal adenocarcinoma (PDAC) belongs to the group of killer human cancers. Its ferocity is sustained by an unusual mix of genetic changes—primarily in KRAS and TP53—a hypoxic as well as desmoplastic tumor microenvironment, plus metabolic and redox adaptations that allow tumor life amidst intense stress situations. **Content**: This paper will discuss the molecular networks of wild-type and mutant p53, wild-type and mutant KRAS, PUMA, TIGAR, PRMT5, NRF2, oxygen tension, reactive oxygen species (ROS), and oxidative stress pathways that contribute to pancreatic cancer. It will describe how these factors help set the tumor’s redox state and control apoptosis and therapeutic resistance. This shall therefore specifically discuss what role oxygen gradients play in pancreatic tissues, as well as retinoic acid, together with redox-targeted therapies that are specific to vulnerabilities within such types of networks. **Summary and Outlook**: An understanding of the crosstalk of these molecular pathways will be critical in designing rational therapeutic strategies. Genetics, metabolism, and microenvironmental integration may open a path toward combinatorial therapies that would resensitize PDAC to apoptosis and overcome resistance to current treatments.

## Simple Summary

Pancreatic ductal adenocarcinoma (PDAC) is a highly lethal cancer characterized by complex genetic mutations and a harsh tumor microenvironment. The disease involves molecular networks driven by key regulators like KRAS and p53, with KRAS mutations initiating uncontrolled growth and p53 loss removing cellular control mechanisms. PDAC’s environment includes severe hypoxia and oxidative stress, where cancer cells adapt redox pathways to survive high reactive oxygen species and promote invasion and therapy resistance. The synergy between KRAS signaling and redox imbalance forms a metabolic shield against conventional treatments, underscoring the need for therapeutic strategies that target this crosstalk and metabolic adaptations to hypoxia.

## 1. Introduction

Pancreatic ductal adenocarcinoma (PDAC) accounts for approximately 90% of neoplasms affecting the pancreas, and at present, it ranks as the sixth leading cause of cancer-related mortality in the world. However, the incidence of this malignancy is projected to progressively increase in the period ranging from 2030 to 2050, becoming the third most common cause of death [1]. This unfavorable scenario is further complicated by the fact that advances in surgical procedures and chemotherapy regimens have not resulted in a significant improvement in the outcome of patients suffering from this malignancy [2]. Their long-term prognosis has remained very poor worldwide over the years, with an estimated 5-year survival rate ranging from 5 to 8% [3,4]. The aggressive biological behavior of this neoplasm is caused by a combination of factors, including progressive, cumulative metabolic alterations in pancreatic cells undergoing malignant transformation, a tissue reaction characterized by dense stromal production, early systemic dissemination, an asymptomatic course with late diagnosis, and resistance to treatment [5]. All these factors allow cancer cells to survive and thrive in an unfavorable, hypoxic environment with reduced nutrient supply and an acidic pH [6,7]. The molecular pattern of PDAC is characterized by mutations developing in the *KRAS*, *TP53*, *CDKN2A*, and *SMAD4* genes [8,9,10,11,12]. In particular, activating changes in *KRAS* are detectable in approximately 90% of patients and represent early events in pancreatic carcinogenesis. About 10% of subjects with PDAC harbor wild-type *KRAS* [13]. These patients generally have a more favorable clinical course and longer survival compared with individuals with mutated *KRAS* [9,10]. On the other hand, changes in *TP53*, detectable in about 50-70% of individuals with PDAC, as well as in the *CDKN2A* and *SMAD4* genes, occur during the invasive and metastatic stages of this malignancy [10,14]. In particular, the interplay between the mutated KRAS (mKRAS), the product of the changed *KRAS* gene, and the mutated p53 (mp53), the protein originating from the changed *TP53* gene, represents a crucial event in pancreatic carcinogenesis [9,15]. The activation of mKRAS promotes pro-oncogenic signaling, whereas mp53 is associated with the reprogramming of metabolism and oxidative stress in cells undergoing malignant transformation [8,13,16]. Overall, these events enable these cells to adapt to the modified chemical, biochemical, and physical conditions developing in their nuclear and cytoplasmatic compartments, as well as in neoplastic pancreatic stroma, during the process of carcinogenesis [17,18]. Malignant cells require elevated levels of biomolecules to survive, grow, and proliferate, including glucose, amino acids, lipids, nucleotides, and energy substrates such as adenosine triphosphate (ATP) [17,18,19]. The supply of these substrates strongly depends on the functional intrinsic changes in a wide series of cancer cells’ activities, including glucose uptake, glycolysis (mainly aerobic glycolysis, also known as the Warburg effect), pentose phosphate pathway, tricarboxylic acid cycle [17,19,20], metabolism of amino acids (in particular glutamine) and lipids [21,22]. These biological processes generate elevated levels of reactive oxygen species (ROS) under low-oxygen tension in pancreatic tissue, which can have potentially harmful effects on cancer cells [23,24]. ROS can both foster and restrain the progression of tumors in general and of PDAC in particular, depending on their type and on their stage of development [23,24,25]. Therefore, malignant cells activate antioxidant systems present in normal cells, such as NRF2 and TIGAR, and promote mitochondrial adaptations to mitigate potential damage and maintain redox balance [26,27,28]. Additional factors are involved in this highly complex molecular landscape, such as PUMA [29], a master regulator of apoptosis, and PRMT5 [30], an enzyme that catalyzes the dimethylation of the amino acid arginine on histone and non-histone proteins in the cell genome. In particular, PUMA is involved in the processes regulating cell survival or death as it controls, via a p53-mediated pathway, the integrity of mitochondrial structure and function [29,31]. A wide range of harmful stimuli may cause injury in biomolecules, DNA, and micro-organelles [26,28]. These events generally trigger the activation of a series of cellular defense systems to repair the damage and restore normal cell activity [26,27,28]. However, if the injury is irreparable, an enzymatic cascade is activated, inducing PUMA-mediated mitochondrial damage with subsequent cell apoptosis [29,32]. On the other hand, PRMT5 modulates chromatin remodeling, RNA maturation, gene transcription, and intracellular signaling cascades [30,33]. Key roles in this complex interaction are played by the oxygen gradient among pancreatic epithelial cells [7,34] and the dense stromal microenvironment [20,34,35]. The normal pancreatic tissue is physiologically characterized by a low-oxygen tension, and one of the crucial PDAC features is the presence of a dense fibrotic stroma. This type of structure further decreases oxygen availability by compressing blood vessels, thereby reducing perfusion [34,35]. Overall, this type of confirmation and spatial orientation both promote selective pressure on malignant cells that develop strong redox control responses, thereby enabling their survival under conditions of vigorous oxidative stress [23,34,35,36]. This microenvironment creates a compartmentalized metabolic network and is strongly involved in PDAC resistance to treatment [17,19,20,21,22,24,36]. Retinoic acid, a compound derived from vitamin A, has been proposed as a potential agent for inducing cell differentiation and modulating redox balance in some malignancies, including PDAC [37]. Preliminary studies have reported that this fat-soluble vitamin exerts several functions, including suppression of cancer-associated fibroblast activities, regulation of ROS levels, and restoration of cell differentiation pathways [37,38]. Several early-phase clinical trials are assessing its use alone or in association with chemotherapy [38,39].

This narrative review examines and summarizes current knowledge on the complex interaction among several players involved in the complex process of pancreatic carcinogenesis, progression, metastasis generation, and response to therapy. These factors, including wild-type or mutant KRAS [16,18,19,28,31,40], wild-type or mutant p53 [8,10,15], PUMA [29,32], TIGAR [26,27,41], NRF-2 [28,40], PRMT5 [30], and Reactive Oxygen Species [23,26,42], normally act as master regulators of a wide series of crucial cell functions, such as metabolism, redox status, motility, proliferation and differentiation [40,41,42]. Our paper also describes other important factors involved in pancreatic carcinogenesis, such as mechanotransduction in cell biology, extracellular matrix organization, stromal stiffness, and viscosity. Furthermore, retinoic acid biology, its potential role as a therapeutic strategy for pancreatic cancer, and its limitations and potential adverse effects in its treatment are widely discussed. Mutations in these players, as well as their dysregulated activities, alter their biological functions, leading step by step to PDAC development.

## 2. The Concept of Malignant Microenvironment in Neoplasms and in Pancreatic Cancer

**Figure 1:**
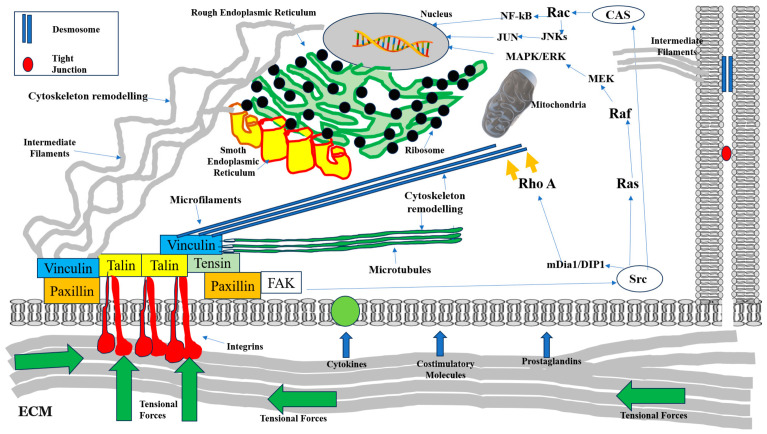
Epithelial cells, found in human tissues, are arranged into continuous, well-ordered, and closely interconnected layers with proper organization and spatial disposition, and adhere to underlying supporting substrates, such as the basement membrane (MB) and ECM. On the whole, these structures are defined as epithelial tissues or epithelia. Epithelial cells interact with one another and with the basement membrane and/or the extracellular matrix through specialized components. In particular, these elements, including desmosomes and tight junctions, connect the plasma membranes of adjacent epithelial cells, and integrin-anchored focal adhesion receptors allow epithelial cells to adhere to the basement membrane and the ECM. Desmosomes (via Intermediate filaments), tight junctions (via actin component of the cytoskeleton), and Integrins (via microfilaments) are bound to the cytoskeleton, a supportive cellular element detectable in the cytoplasm of each eukaryotic cell [43]. On the whole, these components cooperate to form a single functional unit composed of epithelial cells with their intracellular micro-organelles, basement membrane, and ECM. According to the “tensional integrity or tensegrity” model, in tissues of all living organisms, cells with their cytoskeleton and micro-organelles, as well as ECM and connective tissue, represent tensed structures and are subjected to both physical (tensional and compressive forces) and chemical/biochemical stimuli (such as ROS, as well as chemokines, cytokines, costimulatory molecules, and prostaglandins). Integrins connect cells to extracellular compartments. Tensional forces, focused within focal adhesion structures, induce engagement and clustering of these receptors. This event is associated with the recruitment of some signaling proteins, including talin, paxillin, tensin, and alpha-actinin, and promotes a connection with actin cytoskeletal filaments, microfilaments, intermediate filaments, and microtubules, leading to cytoskeleton rearrangement. Furthermore, several enzymes, including Focal Adhesion Tyrosine Kinase (FAK), c-Jun N-terminal Kinase (JNK), mitogen-activated protein kinase (MAPK), mitogen-activated protein kinase (MEK), extracellular signal-regulated kinase (ERK), and the non-receptor tyrosine kinase family, known as Src Family Kinases (Src) and Diaphanous-related formin-1 (mDia1/DIP1), are also concentrated at focal adhesions, where they transfer ECM-originated stimuli to cellular signaling cascades. Several components of the Rho family of small GTPases are additional important enzymes that modulate this complex process. In particular, Src may directly activate Ras, Raf, MEK, MERK, MAPK, or indirectly, via CAS and Rac, nuclear factor kB (NF-kB), JNK, and JUN. On the whole, these enzymes modulate genome transcription and protein synthesis.

Homeostasis and proper function of mammalian tissues depend on maintaining normal cell shape and extracellular matrix (ECM) architecture, as well as adequate dynamic interactions among their constitutive elements. For several years, Researchers have focused their efforts on studying these components at the molecular level, combining chemical, biochemical, physical, and mathematical concepts. A wide range of human tissues contain epithelial cells, arranged into thin, continuous, protective, and closely interconnected layers. These groups of epithelial cells, with well-ordered organization and spatial disposition and associated with underlying supporting structures such as the basement membrane (MB) and ECM, are referred to as epithelial tissues or epithelia. Epithelial cells interact with one another and with the basement membrane and/or extracellular matrix through specialized components. In particular, these elements are represented by desmosomes and tight junctions, which connect the plasma membranes of adjacent epithelial cells, and by integrin-anchored focal adhesion receptors, which allow epithelial cells to adhere to the basement membrane and the ECM. Furthermore, desmosomes, tight junctions, and Integrins are connected with the cytoskeleton, a supportive element detectable in the cytoplasm of each eukaryotic cell [43]. On the whole, these components cooperate to form a single functional unit composed of epithelial cells with their intracellular micro-organelles, basement membrane, and ECM. This very interesting concept was introduced by Ingber et al. In the 1980s, he applied physical and mathematical principles to the study of biological systems and proposed the “tensional integrity or tensegrity” model. According to his hypothesis, in the tissues of all living organisms, cells with their cytoskeletons and micro-organelles, as well as ECM and connective tissues, constitute tense structures and are subjected to both physical and chemical stimuli. The first group of signals includes tensional and compressive forces, whereas the second group comprises chemical compounds, such as reactive oxygen species (ROS), and biochemical mediators and soluble factors, such as chemokines, cytokines, costimulatory molecules, and prostaglandins. Therefore, cells control and determine their biological behavior and fate by modulating and integrating the activities of their metabolic and signal transduction machinery. Cells sense mechanical signals originating both within their microenvironment and in the extracellular compartment, via integrins. These receptors, in association with desmosomes, tight junctions, the cytoskeleton, the basement membrane, and the extracellular matrix, generate a bidirectional pathway through which physical stimuli are transmitted synchronously and ubiquitously to all cells of the tissue and to the underlying supporting structures [44]. Therefore, each tissue and cell, in its context, is subjected to endogenous and exogenous forces. The first class is characterized by forces largely produced by cytoskeletal contractility within the cells, whereas the second one includes a variety of forms, such as gravity, shear stress, tensile, and compressive forces. Cells sense exogenous forces through their interaction with the basement membrane and the ECM. Several local stiffnesses of the ECM are important mechanical effectors of cell behavior. On the whole, this three-dimensional, dynamic macromolecular framework helps stabilize tissue architecture and provides mechanical resistance to deformation. Therefore, changes in the structural components of the connective tissue and of the extracellular matrix (such as modification in type of collagens or of matricellular proteins) as well as in the structure of the cellular microenvironment (changes in the composition of cytoskeletal microfilaments or microtubules), as occurs, for example, in diseases characterized by persistent tissue inflammation, determine an alteration of some biophysical tissue parameters, such as its stiffness, viscosity and geometry [45]. Under normal conditions, the mutual cooperation among cells, their organelles, the ECM, and connective tissue is associated with a self-maintaining biochemical and biomechanical loop. This continuous cycle preserves normal morphology, spatial disposition, and activity of cells embedded in tissues, proper shape and intracellular localization of their micro-organelles, such as the nucleus, mitochondria, rough and smooth endoplasmic reticulum, Golgi apparatus, lysosomes, and cytoskeleton, as well as ECM composition, its three-dimensional arrangement in organs, and its function [46,47]. In recent years, oncologists have focused on these topics as crucial elements in cancer development. Carcinogenesis is a highly complex and dynamic process in which a wide range of cellular and non-cellular components exert cooperative effects to modulate the initiation, growth, progression, and spread of malignancies [48]. Several elements, closely interconnected and forming a self-maintaining loop, are involved in the development of tumors in general and of pancreatic adenocarcinoma in particular. These components are represented by different sets of cells (cells undergoing malignant transformation, immune cells, cancer-associated fibroblasts, i.e., CAFs, endothelial cells/vessels and stromal cells) and their intracelluar elements (cytoskeleton and micro-organuli, such as nucleus, mitocondria, endoplasmic reticulum) as well as soluble mediators, (chemokines, cytokines, costimulatory molecules, reactive oxygen species and prostaglandins) structural- (such as collagens, proteoglycans and glycoproteins) [49], and regulatory proteins (such as matricellular proteins) [50] in concentration and in composition different from those detectable in normal tissues [51] as well as intracellular and dysregulated physical-stimuli (microenvironment stiffness and tensional/compression forces). All these elements, in concert, generate a self-maintaining and supportive dynamic niche in which cancer cells can survive, evade immune system surveillance, proliferate, and spread to distant sites. Recent evidence suggests that niche stiffness promotes and preserves the stemness of malignant stem cells and determines their fate [52]. This concept applies to both primary tumor development and metastatic disease [53,54]. According to our current knowledge, the process of carcinogenesis in general and in PDAC, in particular, are characterized by a large series of intracellular and extracellular modifications. Changes in matrix stiffness and viscosity alter the transcriptome of PDAC cells. The progressive development of genomic mutations in cells that are undergoing malignant transformation is associated with cellular and tissue remodeling, with modification of cytoskeleton composition, structure, and grade of tensional forces, with subsequent changes in intracellular disposition of micro-organuli, such as mitochondria, endoplasmic reticulum, and nucleus. Available studies suggest that mechanical loading is coupled with cellular regulation and that this process is crucial for maintaining tissue homeostasis. An imbalance between these two elements is associated with the development of pathological conditions, including carcinogenesis. Changes in nuclear morphology may determine a remodeling of chromatin conformation with an increase or an inhibition in transcriptional activities of a wide range of genes, with significant modifications in the synthesis and release of structural and non-structural proteins [55]. In particular, stiffness-related signaling cascades modulate epigenetic events, such as DNA methylation, histone modifications, and non-coding RNAs, and influence the synthesis of crucial ECM elements and matrix-modifying enzymes, leading to pathological tissue remodeling and cancer development [56]. Furthermore, malignant cells are characterized by alterations in the activity of mitochondria and other organelles, with significant remodeling and rewiring of numerous functions, including signaling pathways, metabolism, motility, differentiation, and apoptosis [57]. Cancer cells may be regarded as learning structures, able to sense alterations in physical stimuli in their intra- and extracellular microenvironments, such as stiffness-induced mechanical stresses. These changes are stored in a sort of transient or persistent mechanical memory system. Interestingly, the intensity and duration of mechanical stress have important effects in this setting, as long-term, strong stimuli induce irreversible mutations [58]. The introduction of 3D cell culture systems has improved our understanding of these processes with the purpose of mimicking a pathological mechanical environment to understand how malignant cells adapt their metabolism to physical stimuli of the cellular microenvironment. Available studies using these models have demonstrated that some metabolic activities, such as glycolysis, are regulated by the viscoelastic properties of the substrates where cells are cultured, including stiffness and viscosity. In particular, HepG2 cells grown in soft media have exhibited significant mitochondrial membrane depolarization and downregulation of mitochondrially encoded cytochrome c oxidase I, with upregulation of glycolysis [59]. Furthermore, according to current evidence, all malignant tissues, and PDAC in particular, exhibit varying degrees of heterogeneity in structure and spatial arrangement, depending on the distribution of their constituent elements. For example, the different types of collagen and matricellular proteins present in PDAC tissue can differ significantly, both quantitatively and structurally, from those observed in normal pancreatic tissue. This circumstance depends on several factors. These proteins may be synthesized in greater quantities in different, normal polymorphic forms, or they may arise from abnormal transcription or splicing of the genes that encode them. This variability is associated with heterogeneity in stiffness, geometry, viscoelastic properties, and interstitial fluid pressure, even within different areas of the same malignant lesion. Similar considerations can also be proposed for other structural components of tumor tissue. Overall, these characteristics also modulate cellular dormancy and properties of cancer stem cells as well as influence their ability to survive and to disseminate to distant organs [60]. Furthermore, in vitro and in vivo studies support the concept that collagen density and fiber alignment type induce an immunosuppressive microenvironment in PDAC, even in the early phases of carcinogenesis. In particular, increased collagen alignment in histological specimens from patients with this malignancy is associated with a poor prognosis. Macrophagic infiltration of the periductal space adjacent to pancreatic intraepithelial neoplasia (PanIN) is an early event in pancreatic carcinogenesis, and it supports the establishment of a cancer-permissive, immunosuppressive microenvironment. This event is associated with the impairment of the immune response against malignant cells, and it is characterized by the inhibition of T cells’ influx into the tissue, by the prevention of their killing, and by the promotion of tumor growth. It has been reported that PDAC tissue specimens from patients with this disease are richer in collagen (primarily type I collagen) and exhibit increased fiber alignment in the tumor microenvironment, compared with non-neoplastic tissue [61]. Recent studies have proposed a more realistic model, termed “collective migration,” to describe how PDAC cells generate metastases. According to this paradigm, the population of these neoplastic cells may be subdivided into two groups with a different functional hierarchy. In particular, during migration, a group of these cells acts as the leaders, whereas the other cluster acts as the followers. In the first group, cells need more ATP and produce energy through oxidative phosphorylation in comparison with the subset of follower cells. However, the increase in collagen alignment reduces metabolic differences between these two cell subsets, strongly suggesting that the composition and arrangement of extracellular matrix fibers alter tumor cell metabolism [62]. Furthermore, in recent years, researchers have focused their attention on understanding how physical stimuli, primarily stiffness, can impact the structure and arrangement of individual cellular components, including proteins or enzymes (KRAS and p53), receptors, or micro-organelles (mitochondria), in a wide range of malignancies. Currently, few studies have been conducted with this aim, not only in PDAC, but also in tumors affecting other organs. This strategy could be useful for developing a common model for the study not only of PDAC but also of malignancies that arise in sites other than the pancreas. Some researchers have investigated the role that mutations in the *KRAS* gene may exert in the mechanics of cancer cells. Changes occurring in this gene result in altered cytoskeletal rearrangement and actomyosin contractility of neoplastic cells. These modifications, in turn, reprogram the mechanical properties of cells undergoing cancer transformation and alter the modality of interaction with the surrounding mechanical microenvironment. It may also be supposed that the increase in stiffness itself may induce mutations in the *KRAS* gene, and the KRAS-mutated protein, through cytoskeleton remodeling, contributes to promoting malignant transformation of cells. This event generates a self-maintaining mechanical memory loop and contributes to the survival and spreading of cancer cells [63,64]. Furthermore, physical stimuli may also influence p53 intracellular localization and its related functions. In a recent in vitro study, primary murine chondrocytes were cultured on substrates with different stiffness. Cell growth in soft medium (0.5 kPa) has been associated with nuclear translocation of p53. On the other hand, p53 extranuclear localization has been observed in chondrocytes cultured in stiffer substrates [65]. Mitochondria are also strongly involved in the processes of cancer development and spreading. Current evidence suggests that a wide spectrum of stimuli from intracellular and extracellular microenvironments, respectively, including mtROS tone, mtDNA integrity, epigenetic rewiring, cellular stiffness, genome mutations, and oxygen–nutrient supply, ECM mechanics, stromal/inflammatory markers, act in concert and modulate mitochondrial control systems. Therefore, mitochondria regulate cellular homeostasis by driving bioenergetics, redox status, metabolic rewiring, fission–fusion processes, mechanosensitive components, EMT plasticity, and Ca^2+^ balance [66]. Recently, in vitro studies have suggested that breast cancer cells grown on soft substrates exhibit increased levels of F-actin in the peri-mitochondrial regions. Variation in mitochondrial dynamics is associated with increased mitochondrial-induced ROS generation and stimulates NRF2-mediated programs of antioxidant transcriptional response, leading to an increase in cystine uptake and glutathione metabolism. On the whole, these events, such as mitochondrial fission, represent a link connecting processes of mechanotransduction in the microenvironment to cellular metabolism and redox status, as well as a significant impact on metastases generation and chemotherapeutic resistance (Figure 1) [67]. Taking advantage of all this evidence, we have examined several processes involved in the development of PDAC.

## 3. KRAS and TP53 Role in PDAC: Signaling and Metabolic Rewiring

Activating mutations in the *KRAS* oncogene are observed in the majority of PDACs, mainly at codon 12 [8,13,68]. This alteration represents the earliest and most frequent event during the dynamic process, leading to the occurrence of pancreatic cancer [6,7,8,9]. In normal pancreatic cells, the KRAS protein switches between an inactive “off” state (bound to GDP) and an active “on” state (bound to GTP) [8,13,68]. This complex controls the activity of some signaling pathways, including the RAF–MEK–ERK, MAPK, and PI3K–AKT–mTOR cascades, and promotes fine-tuned modulation of crucial cell functions, such as metabolism, proliferation, differentiation, motility, survival, and anticancer treatments [69,70,71].

The mutated KRAS (mKRAS) protein is locked in an active status, and this event constitutively induces the activation of its GTPase function. This event prevents efficient hydrolysis of GTP back to GDP, leads to the persistent induction of gene transcriptional programs, and promotes malignant transformation [68,72].

### 3.1. KRAS Mutation and Metabolic Reprogramming

The ability of the mKRAS protein to rewire cellular metabolism of glucose, lipids, and amino acids is one of the distinctive features of pancreatic carcinogenesis [17,18,19,20,21,73]. Malignant pancreatic cells exhibit a switch in the anabolic and catabolic programs of these biomolecules with an increase in glucose uptake, preferential aerobic glycolysis (known as the Warburg effect), activation of the enzymatic cascade, as well as conversion of pyruvate into lactate even in the presence of normal oxygen tension levels within tissue and cells [17,18,19,20,21,26,28,73,74]. This metabolic reprogramming allows cancer cells to shunt substrates generated by glycolysis toward the biosynthesis of crucial metabolites, such as nucleotides, amino acids, and NADPH, via the pentose phosphate and serine/glycine pathways [75,76]. Furthermore, the mKRAS protein modulates glutamine metabolism via a non-canonical cascade [18,19]. Specifically, in normal cells, glutamine is generally converted to α-ketoglutarate by glutamate dehydrogenase [21,22], whereas in malignant pancreatic cells, it mainly activates the aspartate transaminase (GOT1) pathway [21,77]. The activation of this enzymatic cascade generates large amounts of NADPH [20,78,79]. The increased levels of this cofactor contribute to inducing an antioxidant status in malignant cells and tissues. By counteracting ROS generation, this event promotes their survival and spreading (see paragraph 3 “*The complex interplay among Reactive Oxygen Species, NRF2, and TIGAR in metabolic adaptation of malignant pancreatic cells*”). This product plays a crucial role in counteracting ROS and in preserving antioxidant status in malignant cells and tissues. This event is essential for promoting tumor survival and its spreading [80,81].

In addition to glycolysis upregulation, the mKRAS protein also modulates mitochondrial activity [82].

### 3.2. Wild-Type p53: A Metabolic Master Regulator

The wild-type form of p53, a crucial regulatory protein, acts as a master tumor suppressor by counteracting many of the metabolic functions stimulated by mKRAS [10,15,83,84]. In particular, a wide range of genes controlling key cellular processes, such as glycolysis (e.g., TIGAR), the mitochondrial respiratory chain and oxidative phosphorylation [19,20,21,85,86], antioxidant defense, and ROS production, are transcriptionally modulated by p53 [15,87]. Overall, this regulatory protein helps maintain cellular homeostasis by preventing excessive activation of these pathways [15,88].

Furthermore, p53 induces upregulation of PUMA and other pro-apoptotic genes in response to metabolic or oxidative stress [15,29,32,89]. In physiological conditions, these events lead to apoptosis in cells experiencing damaged mitochondria or excessive ROS production [90,91] (see paragraph 3 “*The complex interplay among Reactive Oxygen Species, NRF2, and TIGAR in metabolic adaptation of malignant pancreatic cells*”). Overall, these tightly regulated processes ensure that cells undergoing activation of pro-oncogenic programs and critical metabolic alterations are either repaired or eliminated, thereby preventing cancer transformation [19,20,21,28,92].

### 3.3. KRAS–p53 Cooperation in Tumor Progression

**Figure 2:**
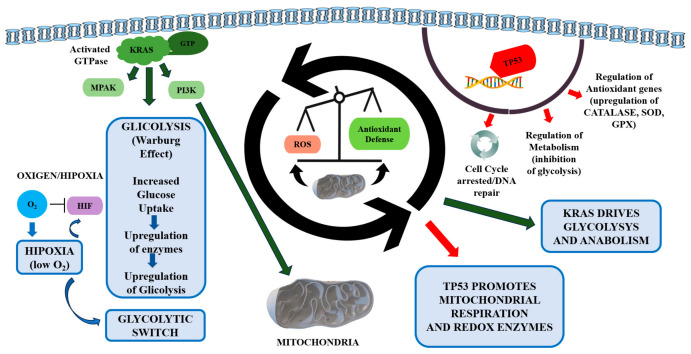
Some of the crucial events arising in pancreatic cells during the process of carcinogenesis. The majority of PDACs exhibit activating mutations in the *KRAS* oncogene, mainly at codon 12. In normal pancreatic cells, the KRAS protein controls the activity of some signaling pathways, such as RAF–MEK–ERK, MAPK, and PI3K–AKT–mTOR cascades, involved in the regulation of some key cell functions, such as metabolism, proliferation, differentiation, motility, survival, and anticancer treatments. Mutations in the *KRAS* gene are associated with the generation of an altered protein (mKRAS) that is constitutively active. This condition causes persistent stimulation of the above-reported enzymatic pathways and promotes malignant transformation. In particular, the mKRAS protein rewires cell metabolism of glucose, lipids, and amino acids, inducing an increase in glucose uptake, preferential aerobic glycolysis (known as the Warburg effect), activation of the enzymatic cascade, and conversion of pyruvate into lactate even in the presence of normal oxygen tension levels within tissue and cells. This metabolic reprogramming allows cancer cells to shunt glycolytic substrates toward the biosynthesis of key metabolites, including nucleotides, amino acids (such as glutamine), and NADPH, via the pentose phosphate and serine/glycine pathways. Mutated-p53 (mp53) stimulates NRF2 activity through the disruption of its negative modulatory function or through NRF2 target gene expression. The activation of the KRAS–p53–NRF2 axis counteracts ROS generation and promotes cell survival and proliferation under conditions of metabolic and hypoxic stress. Furthermore, during carcinogenesis, pancreatic cells accumulate mutations in the *TP53* gene. Wild-type p53 protein is a master tumor suppressor, as it counteracts a large series of metabolic cell functions stimulated by mKRAS, including glycolysis, mitochondrial respiratory chain, oxidative phosphorylation, antioxidant defense, and ROS generation. In normal conditions, this regulatory protein prevents abnormal activity of these cascades, upregulating PUMA and other pro-apoptotic genes in response to metabolic or oxidative stress. Therefore, damaged cells undergo apoptosis, thereby preventing cancer transformation. During pancreatic carcinogenesis, KRAS- and p53-mutated proteins cooperate in the development of this malignancy. In particular, the constitutive activity of the mKRAS protein is an early event in PDAC onset, as it induces metabolic rewiring and cell proliferation. On the other hand, the mutated p53 protein eliminates the apoptotic checkpoint and promotes a metabolically favorable microenvironment for malignant cells, enabling them to tolerate the elevated ROS amounts generated during rapid cell proliferation, enhancing their aggressiveness and making them resistant to anticancer therapies.

The cooperative interplay between mKRAS and p53 is a crucial axis in the onset and development of PDAC [15,93,94]. Whereas the constitutive activity of the mKRAS protein represents the starting point in the process of pancreatic carcinogenesis as it causes metabolic rewiring and proliferation of cells [24,28], mutated p53 removes the apoptotic checkpoint and establishes a metabolically favorable microenvironment for cancer cells [15], increasing their aggressivity and making them resistant to anticancer treatment [95,96] (Figure 2). Studies in mouse models have shown that the *KRAS* mutation alone can induce PanIN formation, and that the progression to invasive malignancy occurs in the presence of the *TP53* mutation [97,98]. This tandem interaction between the two proteins encoded by the KRAS [16,18] and TP53 [8,10,15] genes induces a series of crucial metabolic effects [99]. Mutant p53–mediated antioxidant responses promote KRAS protein-mediated glycolysis and glutamine metabolism [100]. All these events enable cancer cells to tolerate the elevated levels of ROS generated during rapid cell proliferation [101,102]. The interplay between mKRAS and p53 forms an axis characterized by elevated glycolytic flux, preserved redox balance, and flexible mitochondrial function, enabling survival of PDAC cells under harmful conditions [16,103].

## 4. The Complex Interplay Among Reactive Oxygen Species, NRF2, Mitochondria, and TIGAR in Metabolic Adaptation of Malignant Pancreatic Cells

In biological systems, ROS are involved in the modulation of several essential physiological intracellular processes, such as energy production, mitoptosis, autophagy, molecule synthesis, proliferation, apoptosis, migration, adhesion, energy metabolism, and cytoskeletal rearrangements, as well as extracellular events, including immune response, inflammation, fibrosis, and tissue repair. As previously reported, these chemical mediators, by acting as second messengers in interactive cooperation with physical stimuli, regulate the activity of a large series of cell signaling cascades, such as mitogen-activated protein kinases (MAPK)s and phosphoinositide 3-kinase (PI3K)/protein kinase B (Akt) as well as transcription factors such as nuclear factor erythroid 2-related factor 2 (NRF2), hypoxia-inducible factor 1α (HIF-1α), activator protein 1 (AP-1) and nuclear factor kappa-light-chain-enhancer of activated B cells (NF-κB). The acronym ROS defines a framework of highly reactive oxygen-derived molecules, including both free radical and non-radical elements. The first group includes superoxide anion (O_2_•) and the perhydroxyl radical (HO_2_•), the hydroxyl radical (HO•), the peroxyl radical (ROO•) and the alkoxyl radical (RO•), whereas hydrogen peroxide (H_2_O_2_), singlet molecular oxygen (1 O_2_), hypochlorous acid (HOCl), and organic hydroperoxides (ROOH) are enclosed in the second one [104]. Given their chemical and physical properties, they can interact with crucial macromolecules both within and outside the cells, such as ribonucleic and nucleic acids, lipids, structural and non-structural proteins, resulting in oxidative stress and irreversible cell—as well as tissue—damage and triggering programmed cell death through necroptosis, apoptosis, or other pathways. Therefore, normal mammalian cells have evolved tightly regulated mechanisms to safeguard their redox status by controlling the balance between ROS synthesis and removal. The generation of reactive oxygen-derived molecules during normal cellular activities occurs in concert with the activation of cellular antioxidant systems, which serve as defenses at the cellular and tissue levels. In this intricate context, ROS themselves also play a crucial role in cancer biology, exhibiting dual function. These chemical compounds may act as both tumor-promoting and tumor-suppressive mediators, depending on several factors, including their concentrations in the neoplastic microenvironment. In particular, low levels of ROS promote carcinogenesis by stimulating malignant cells to divide, migrate, invade adjacent areas, or metastasize to distant sites and develop drug resistance. On the other hand, high levels of these chemical mediators damage organelles and membranes, triggering programmed cell death [105].

Therefore, tumor cells have to strictly regulate their oxidant and antioxidant processes to maintain ROS production at proper levels, thus ensuring their survival in a microenvironment, compromising metabolism and oxygen supply [16,103,106,107]. ROS are produced mainly via mitochondrial oxidative phosphorylation, involving NADPH oxidases, under both physiological conditions and oncogene-driven metabolic processes [108,109]. Moderate ROS levels act as signaling molecules, promoting proliferation and adaptation [110]. However, the excessive ROS production generally causes oxidative damage, senescence, or apoptosis [85,111]. Therefore, PDAC cells have developed sophisticated mechanisms to buffer ROS without eliminating their growth-promoting signaling roles [26,27,28,29,112]. Mitochondria play a crucial role in PDAC cells, as they are involved in both anabolic processes, supplying intermediate metabolites for lipid, amino acid, and nucleotide production, and ROS generation, thereby regulating oncogenic pathways [113,114]. Mitochondria remain functional and play key roles in both the biosynthesis of substrates for rapid malignant cell proliferation and the regulation of their redox status, rather than in ATP generation [115,116,117]. This “hybrid metabolism” further promotes PDAC cell survival under fluctuating oxygen tension [118].

### 4.1. ROS Generation in Pancreatic Cancer

The most important effect of the mKRAS protein is the development of a hypermetabolic state, characterized by increased glycolysis, glutamine metabolism, and mitochondrial activity [18,19,20,21,119,120]. Furthermore, although glycolysis is the primary pathway for energy production, oxidative phosphorylation in mitochondria remains active and is a major source of ROS [121,122]. Mutant KRAS disrupts mitochondrial activities by enhancing mitochondrial biogenesis and by preventing mitophagy [113,123,124]. These events promote mitochondrial hyperactivity, which makes mitochondria stress-tolerant [125,126]. Mutant p53 also enhances mitochondrial adaptation to the malignant microenvironment by increasing the transcription of genes that regulate ROS detoxification and mitochondrial metabolism [127,128]. All these events ensure the survival of pancreatic cancer cells in the hypoxic, nutrient-poor, and unfavorable dense stromal tissue [20,115,129]. The dense, hypovascularized PDAC microenvironment is characterized by fluctuating oxygen tension levels [34,35,36,130]. A similar tissue pattern results in increased mitochondrial ROS production during intermittent hypoxia and reperfusion [23,24,74,131]. Furthermore, NADPH oxidases (NOX), particularly NOX4, are upregulated in PDAC cells, thereby promoting the synthesis of superoxide and hydrogen peroxide [21,75,76,77,117,132,133]. These ROS sources, in association with alterations in redox enzyme expression, generate a dynamic redox microenvironment [24,42,134,135]. Malignant cells have evolved tightly controlled mechanisms to reduce or buffer ROS levels, thereby preventing cell death [24,42,136,137].

### 4.2. NRF2: A Master Regulator of Antioxidant Responses

**Figure 3:**
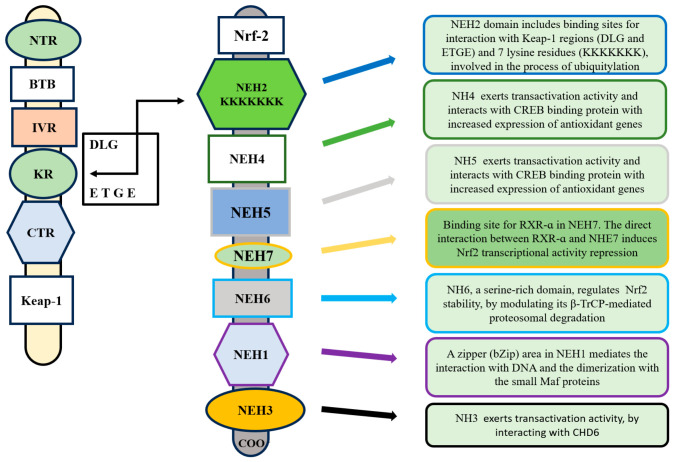
Structure of NRF2 and KEAP1. Nrf2 includes seven NEH components, each of which exhibits distinct activities. The NEH1 domain consists of a basic leucine zipper (bZip) area, mediating both the interaction with DNA via basic region and the dimerization with the small Maf proteins (musculoaponeurotic fibrosarcoma proteins are basic region leucine zipper-type transcription factors) via the bZip motif. The NEH2 domain includes the DLG and ETGE regions, by which it interacts with KEAP1 and seven lysine residues (K), involved in the process of ubiquitylation. NEH3, NEH4, and NEH5 domains exert transactivation activities. In particular, NEH3 interacts with CHD6 (a chromo-ATPase/helicase DNA-binding protein), whereas NEH4 and NEH5 include binding sites for CREB binding protein (CBP). The interaction among these elements increases the expression of antioxidant genes. NEH6 region is a serine-rich region. It regulates NRF2 stability by modulating its β-TrCP-mediated proteasomal degradation (beta-transducin repeat protein). NHE7 includes a binding site for RXR-α. The direct interaction between RXR-α and NHE7 induces NRF2 transcriptional activity repression. Overall, NRF2 regulates the expression of key components of the glutathione (GSH) and thioredoxin (TXN) antioxidant systems and of enzymes, thereby modulating NADPH regeneration, ROS production, xenobiotic detoxification, and heme metabolism. Nrf2 promotes NADPH production via the activation of key NADPH-generating enzymes: glucose-6-phosphate dehydrogenase (G6pd), 6-phosphogluconate dehydrogenase (Pgd), isocitrate dehydrogenase 1 (Idh1), and malic enzyme 1 (Me1). Keap-1 consists of five regions, including the N-terminal region (NTR), Broad-Complex Tramtrack and bric-à-brac (BTB) domain, the intervening region (IVR), double glycine repeats (DGR or Kelch domain), and the C-terminal region (CTR). The BTB domain interacts with Keap1 and promotes binding to IVR with Cul3/RBX1 complex. Under normal conditions, the binding of Nrf-2 to Keap1 in the cytoplasm induces the formation of a ubiquitin ligase complex, resulting in the proteasomal ubiquitin-dependent degradation of NRF2, while Keap1 becomes available again. After oxidative stress, Nrf2 dissociates from Keap1, enters the cell nucleus, and induces the expression of antioxidant genes.

Nuclear factor erythroid 2-related factor 2 (NRF2) is a transcription factor modulating the expression of approximately 200 genes with antioxidant and cytoprotective functions [28,138,139,140,141,142] (Figure 2 and Figure 3). The majority of these genes encode enzymes involved in glutathione production, thioredoxin metabolism, and detoxification [138,139,140,141]. Under normal conditions, NRF2 associates with KEAP1 in the cytoplasm and undergoes proteasomal degradation [139,140,143,144]. Under oxidative stress, NRF2 dissociates from KEAP1, translocates to the nucleus, binds to the transcription-dependent antioxidant response element (ARE), and upregulates the synthesis of enzymes involved in the antioxidant response [139,140,143,144]. In patients with PDAC, NRF2 pathway activity is generally increased in comparison with normal pancreatic cells [143,145,146]. This event may be driven by various mechanisms, such as mutations in NRF2, which promote tumor survival by enhancing cellular antioxidant capacity [146,147]. This occurrence enables malignant cells to counteract the persistent oxidative stress, detectable in the cancer microenvironment [146,147]. In particular, NRF2 activity is a key and required event in KRAS-induced tumors to preserve redox homeostasis and promote tumor growth; genetic ablation of NRF2 prevents the development of PDAC in experimental mouse models [148,149].

Mutated-p53 (mp53) stimulates NRF2 activity through the disruption of its negative modulatory function or through NRF2 target gene expression [148,150]. The induction of the KRAS–p53–NRF2 pathway supports pancreatic malignant cells with strong adaptive mechanisms [28,151,152]. The activation of this axis counteracts ROS production and promotes cell survival and proliferation under metabolic and hypoxic stress [153,154].

### 4.3. TIGAR: Modulator of Glycolysis and ROS

TP53-induced glycolysis and apoptosis regulator (TIGAR) represents a further key master metabolic regulator downstream of p53 [26,27,155]. TIGAR exhibits a fructose-2,6-bisphosphatase activity and exerts a wide series of functions [156,157,158]. This enzyme decreases fructose-2,6-bisphosphate levels, suppresses glycolysis, and shifts glucose-6-phosphate into the pentose phosphate pathway (PPP) [155,159]. This diversion of intracellular metabolism enhances NADPH generation, strengthens antioxidant defenses, and lowers ROS within cells [41,159,160]. In normal cells, wild-type p53-induced TIGAR expression plays a key role in protecting against oxidative stress and preserving metabolic balance [26,27,161]. Furthermore, the dynamic modulation of ROS production by TIGAR is a crucial event in pancreatic carcinogenesis, both at initiation and during progression, and multiple mechanisms contribute to this complex scenario [26,27,41,161,162]. In particular, oncogenic KRAS protein and mutated p53 increase TIGAR levels and upregulate its activity. TIGAR forms a tightly regulated axis with Kras protein and mutated-p53, modulating ROS generation [15,18,21,41,161,162]. According to experimental mouse and human models, levels of TIGAR expression fluctuate as PDAC arises and develops, depending on different phases of carcinogenesis [26,27]. As a consequence, the amounts of ROS in the pancreatic microenvironment also oscillate with different effects on the initiation and progression of pancreatic cancer [155,161]. In particular, TIGAR increases levels that support cancer cell survival and proliferation, as well as the development of premalignant lesions in the early stages of pancreatic carcinogenesis [155,162]. On the other hand, the levels of this enzyme decrease in advanced stages of PDAC development [163,164]. Increased TIGAR levels have been described in the PDAC microenvironment and are associated with elevated PPP flux, ROS scavenging, and resistance to apoptosis [26,27,41]. Furthermore, TIGAR functions in concert with NRF2, and both downstream cascades converge on the generation of NADPH and glutathione [26,27,163,165]. As a consequence, an epigenetic and metabolic autoregulatory loop between NRF2 and TIGAR emerges, generating an antioxidant network that enables malignant cells to preserve their redox balance even under severe oxidative conditions and is involved in PDAC resistance to chemotherapeutic treatments [166,167].

### 4.4. ROS Buffering and Resistance to Therapy

**Figure 4:**
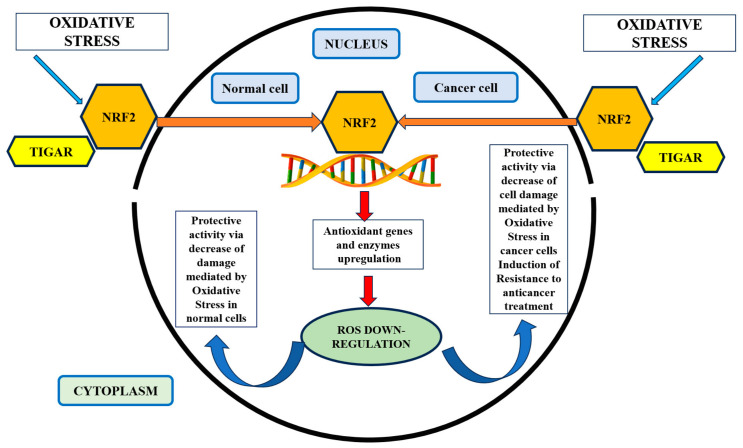
Mechanisms associated with resistance to anti-cancer therapy. The TP53-induced glycolysis and apoptosis regulator (TIGAR)-mediated epigenetic autoregulatory loop contributes to the development of therapeutic resistance in malignant cells. TIGAR and NRF2 are two key members of this network, as they control the transcription of a large series of antioxidant genes. Under oxidative stress, both elements, normally detectable in the cytoplasm, interact. This process is associated with NRF2 translocation into the nucleus, and it leads to the synthesis of antioxidant enzymes. Overall, this autoregulatory loop plays a protective role in normal cells undergoing oxidative stress, limiting ROS production and thereby attenuating injury. The activation of this network in malignant cells induces a significant decrease in ROS synthesis, prevents cancer cells’ death, and promotes resistance to anti-cancer treatment.

The increase in antioxidant capacity mediated by NRF2, TIGAR, and metabolic remodeling has profound implications for the development of effective therapeutic strategies against pancreatic malignancy [167,168,169,170,171] (Figure 4). Current conventional pharmacological treatments (e.g., gemcitabine) and radiotherapy exert their antineoplastic activity, in part, by inducing oxidative damage [172]. This process generally causes cancer cells to die. PDAC exhibits the ability to buffer ROS generation, thereby promoting innate resistance to these treatments [173]. Furthermore, NRF2 activation can induce drug-metabolizing enzymes and efflux pumps, thereby decreasing the intracellular concentration and efficacy of anticancer drugs [174,175]. Redox homeostasis targeting has been proposed as a therapeutic approach for the treatment of PDAC. The most studied strategies include inhibition of glutathione synthesis, suppression of NRF2 signaling, or increasing ROS generation beyond the buffering capacity of cells [176,177]. These approaches require an adequate, well-defined target to prevent injury to normal tissues. Therefore, a proper understanding of the ROS–NRF2–TIGAR network is essential for designing studies that evaluate the efficacy of combination therapies for the treatment of pancreatic cancer [143,178].

## 5. PUMA- and PRMT5-Mediated Regulation of Mitochondrial Function in PDAC

In pancreatic ductal adenocarcinoma (PDAC), a very fine balance exists between malignant cell survival and apoptosis [91,94,95,96,176], which is modulated by several factors, such as oncogenic signaling, redox adaptation, regulators of mitochondrial integrity, and epigenetic control [16,97,98,99,100,101,102,179]. Two crucial elements are involved in this complex network: PUMA (p53 upregulated modulator of apoptosis) [27,91,178] and PRMT5 (protein arginine methyltransferase 5) [30,180]. Both these players interact with the KRAS–p53–ROS pathway and modulate cell fate, mitochondrial metabolism, and PDAC resistance to chemotherapeutic drugs [181,182].

### 5.1. PUMA: p53-Mediated Mitochondrial Apoptosis

PUMA is a component of the BCL-2 family and exerts a highly efficient pro-apoptotic activity [90,183]. In the presence of intracellular stress conditions, wild-type p53 at the transcriptional level induces the upregulation of PUMA [184]. This mediator binds anti-apoptotic factors, such as BCL-2 and BCL-XL [185,186], and counteracts their inhibitory function, inducing the release of some pro-apoptotic proteins such as BAX and BAK [185,187]. Overall, these events induce mitochondrial outer membrane permeabilization, cytochrome c leakage, and caspase activation [185,186,187].

In normal pancreatic cells, PUMA acts as a crucial defense against oncogenic stimuli [188]. Excessive ROS generation, metabolic derangement, or DNA injury caused by abnormal KRAS signaling can stimulate the p53-mediated expression of PUMA, triggering the apoptosis of damaged cells [189]. Nevertheless, in PDAC, this safeguard activity is frequently overcome. The following mechanisms are involved in this process: *TP53* mutation, epigenetic silencing of PUMA, or abnormal high expression of anti-apoptotic proteins [190]. As a result, cells harboring mutated KRAS and elevated ROS amounts do not undergo apoptosis but survive and promote cancer progression [184]. PUMA may also be activated via a ROS-dependent signaling cascade, independently of the p53-mediated induction [191]. Furthermore, PUMA may be stimulated by the upregulation of JNK and other transcription factors [192,193]. This event links intracellular redox stress to mitochondrial apoptosis [194,195].

In PDAC, this pathway is often downregulated by the constitutively increased NRF2 activity, with its antioxidant function [194]. This event prevents ROS-induced death of malignant cells [196].

### 5.2. PRMT5: Epigenetic and Metabolic Regulator

PRMT5 is an arginine methyltransferase [179]. It exerts its activity via the dimethylation of arginine residues in histones and non-histone proteins and plays a crucial role in several cellular processes, such as transcriptional regulation, splicing, DNA repair, and metabolism [197]. PRMT5 is frequently upregulated in PDAC, and its overexpression is associated with a poor outcome in patients suffering from this malignancy and with resistance to current chemotherapeutic regimens [198,199]. PRMT5 affects PDAC biological behaviour via multiple mechanisms [200], such as: (1) repression of genes mediating tumor suppression via histone methylation and induction of apoptotic and cell cycle regulatory cascades silencing [33]; (2) modulation of the oxidative stress status in malignant cells via methylation of crucial transcriptional regulatory factors, this event causes the expression of several antioxidant genes and promotes cancer cells survival under conditions of redox stress [201]; (3) regulation of mitochondrial metabolism, by influencing the activity of genes involved in oxidative phosphorylation and ROS detoxification [202].

Recent reports suggest that in KRAS-mutated PDAC cells, PRMT5 plays a crucial role in maintaining redox homeostasis and mitochondrial activity [203]. Therefore, PRMT5 is a particularly promising potential target for the treatment of this malignancy [204,205]. PRMT5 inhibition enhances mitochondrial ROS generation, promotes PUMA expression, and sensitizes PDAC cells to apoptotic stimuli [206,207,208].

### 5.3. Mitochondrial Activity and Metabolic Flexibility

Although aerobic glycolysis (known as the Warburg effect) represents one of the most important metabolic processes through which PDAC cells synthesize ATP for their energy needs and substrates for their proliferation [209,210], additional intracellular signaling and micro-organelles play a crucial role in supporting the biosynthesis of these compounds, in maintaining redox balance, and in ensuring the survival of neoplastic cells [211,212,213]. PUMA serves as a crucial checkpoint in this network. When redox stress exceeds the buffering capacity, or when mitochondrial activity is altered, PUMA-driven apoptosis is triggered [189,190]. However, in the presence of mutated p53 [13,182], overstimulated NRF2 [25,135,155], and overactivated PRMT5 [166,169,170], this control checkpoint is overwhelmed, leading to persistent mitochondrial stress [29,82,192,214]. This event prevents the elimination of malignant cells via apoptosis [215].

### 5.4. Therapeutic Implications

The interplay among PUMA, PRMT5, and mitochondrial activity is a vulnerability element for PDAC and can be exploited for therapeutic purposes [216,217]. Current studies are exploring strategies to treat this malignancy effectively. Therapeutic approaches that restore PUMA activity, suppress PRMT5 function, or impair redox adaptation in mitochondria are under active consideration in experimental and clinical studies to overcome resistance of PDAC cells to anticancer agents [29,90,218,219]. For example, treatment schedules that combine PRMT5 inhibitors with ROS-inducing drugs have been shown to be effective in KRAS-mutant malignancies [218,220,221]. Furthermore, restoration of normal p53 function or pharmacologic activation of PUMA can sensitize PDAC cells to chemotherapy and oxidative stress [222,223].

## 6. Oxygen Tension in Pancreatic Cells and Malignant Stroma

The presence of a dense stromal reaction (desmoplasia) is one of the most important structural characteristics, detectable in the PDAC microenvironment [20,115,116,224]. The change in the composition of malignant pancreatic tissue is associated with the alteration of its oxygenation and nutrient intake [85,118,225]. In particular, a heterogeneous distribution and strongly reduced oxygen tension are among the most evident characteristics of the microenvironment of pancreatic cancer [82,226]. This event has a significant impact on metabolic adaptation, ROS generation, response to treatment, as well as cellular interplay between malignant cells and stroma [85,110,111,112,120,227,228].

### 6.1. Baseline Oxygen Tension in Normal Pancreatic Tissue

In a healthy pancreas, a relatively elevated oxygen tension is detectable in the exocrine acinar regions, whereas a lower one is observed in the ductal epithelium [85,110,111,112,120]. This pattern is consistent with physiological variations in vascularization and metabolic activity across the different tissue compartments of the organ [229,230]. Pancreatic epithelial cells meet their energy needs via oxidative phosphorylation under normoxic conditions and preserve their redox homeostasis [85,86,87,88,89] via a tightly regulated balance between production of mitochondrial ROS and activation of antioxidant systems [230,231]. Stromal oxygen afflux is finely modulated by a rich capillary network, thereby providing sufficient oxygen to support both endocrine and exocrine activities [35,232,233].

### 6.2. Hypoxia and Oxygen Gradients in PDAC

The process of pancreatic carcinogenesis is characterized by the extensive deposition of a desmoplastic stroma, consisting of fibroblasts, extracellular matrix (ECM), immune cells, and a poor vascularization pattern [78,80,116,234,235]. The composition and spatial disposition of this network in PDAC is associated with a remarkable decrease in oxygen levels [25], with the detection in both preclinical studies and patient samples of oxygen tension within the cancer equal to 1–2% (7–15 mmHg), in comparison with 5–7% in normal pancreatic tissue [34,236,237]. It is well known that pancreatic stromal cells enhance collagen synthesis in response to hypoxic conditions. This event promotes the formation of fibrotic tissue and hampers immune cell recruitment, thereby inducing resistance to chemotherapy and radiotherapy [224,230,231].

Furthermore, oxygen levels are heterogeneously distributed in the different regions of malignant pancreatic lesions [118,136,238,239]. Perivascular compartments are characterized by the maintenance of moderate oxygenation [159,165,166,239,240]. On the other hand, severe hypoxemia is observed in the innermost regions of PDAC, particularly in the stroma, where Cancer-Associated Fibroblasts (CAFs) are often detected in low-oxygen tension areas, and this condition affects their metabolism, extracellular matrix deposition, and paracrine pathways [35,241,242].

### 6.3. Effects of Hypoxia on PDAC Metabolism and ROS Generation

Hypoxia strongly modulates PDAC metabolism via stabilization of hypoxia-inducible factors (HIFs) [243,244]. This event induces transcriptional reprogramming in tumor cells and promotes the processes of glycolysis, proliferation, survival, and angiogenesis under hypoxic conditions [245,246]. In pancreatic cancer cells, stabilization of HIF-1α further increases glycolytic flux and non-canonical glutamine metabolism, already increased by *KRAS* mutation [247,248]. This metabolic rewiring enables malignant cells to survive despite a persistent oxygen-poor supply [20,78,92,249]. Furthermore, hypoxia can be associated with increased ROS generation within mitochondria due to decreased efficiency of the electron transport chain, intermittent phases of tissue reperfusion, and the promotion of oxidative bursts [250,251]. ROS act as signaling regulators that activate NRF2 and other antioxidant cascades, reinforcing and promoting cancer adaptation [252,253]. Induction of these pathways leads to the generation of antioxidant enzymes and molecules that buffer redox stress in the tumor epithelium [254,255,256,257]. Cancer-associated fibroblasts (CAFs) [20] adapt to decreased oxygen tension through the upregulation of glycolysis and the secretion of several substrates, such as lactate, pyruvate, and alanine [258,259,260,261,262].

### 6.4. Oxygen Levels in the Stroma: Interactions Among Fibroblasts and Immune Cells

As in other types of human malignancies, a dynamic interaction also exists in PDAC among stroma and epithelial tumor cells via an oxygen-dependent metabolic crosstalk [253,254,255,256,257]. Cancer-associated fibroblasts (CAFs) [20] adapt to decreased oxygen tension through the upregulation of glycolysis and the secretion of several substrates, such as lactate, pyruvate, and alanine [259,260]. The metabolic pathways of these compounds are highly interconnected [258]. These molecules serve as alternative fuel sources for tumor cells, providing metabolites to meet their energy needs and to support the biosynthesis of substrates essential for their survival and proliferation [19,31,261,262]. This type of metabolic pattern remodeling enables cancer cells to preserve mitochondrial metabolism and redox balance even in hypoxic regions of malignant tissue [7,253,254,263,264].

Furthermore, immune cells, such as Tumor-Associated Macrophages (TAMs) and T cells in the hypoxic stroma [17,265], detectable in PDAC, undergo metabolic and functional rewiring in response to low oxygen levels [19,31,180,266]. Hypoxia tends to induce an immunosuppressive microenvironment in cancer tissue [1,6,7] via several mechanisms, such as HIF stabilization, upregulation of adenosine pathways, and impairment of cytotoxic T-cell activity [267,268,269]. Together, all these factors contribute to tumor immune evasion and resistance to anticancer treatments [1,269,270].

### 6.5. Implications for Therapy

The condition of hypoxia and oxygen-heterogeneous distribution in pancreatic cancer tissue is associated with significant challenges for its treatment [7,268,269,270]. The rationale for the use of radiotherapy in the treatment of human malignancies is based on the assumption that oxygen generates DNA-damaging free radicals, leading cancer cells to death [7,268,269,270,271,272]. However, hypoxic PDAC tissue is generally radioresistant [103,273,274]. Furthermore, a poor vascularization network and high-density ECM hamper the influx of chemotherapeutic drugs to malignant pancreatic lesions, decreasing drug penetration into these sites [274,275,276]. Several therapeutic strategies focused on modifying oxygen tissue levels or on modulating metabolism in hypoxic pancreatic cancer tissue have been assessed [277,278]. These approaches include the potential usefulness of hypoxia-activated prodrugs [279], HIF inhibitors [280], stromal remodeling pharmacological agents [281,282], and metabolic treatments to discontinue stromal–epithelial crosstalk [283]. Retinoic acid, discussed in Section 6, has shown promising activities in normalization of stromal function [284] and in modulation of oxygen-dependent signaling, potentially increasing the probability of response to anticancer treatment [285,286].

## 7. Retinoic Acid: Modulation of Microenvironment Composition, Redox Status in Pancreatic Cancer

Retinoic acid (RA), a derivative of vitamin A, has been shown to act as a pleiotropic modulator of several crucial cell functions, such as differentiation, redox homeostasis, and stromal signaling in PDAC [287,288,289]. Although RA use in clinical practice for solid tumors has historically been limited [219,290], several studies are clarifying its possible role in the modulation of biological processes within the tumor microenvironment (TME) and in regulating its metabolic adaptation. These reports are promoting a renewed interest in RA as a potentially effective adjuvant drug for the treatment of PDAC [291,292,293].

### 7.1. Retinoic Acid Signaling in Normal Pancreas and in Pancreatic Cancer

In the normal pancreas, RA signaling modulates epithelial cell differentiation, acinar-to-ductal cell ratio, and stromal homeostasis via the induction of nuclear retinoic acid receptors (RARs) and retinoid X receptors (RXRs) [294,295]. RAR/RXR heterodimers interact with retinoic acid response elements (RAREs) in gene promoters, controlling the transcription of genes involved in cell differentiation, cell cycle arrest, and redox homeostasis [294,296,297].

In pancreatic cancer, a profound alteration of retinoid signaling is often detectable. Reduced expression of cellular retinoic acid-binding proteins (CRABPs) and of RA in the cancer tissue is associated with loss of epithelial cell differentiation and induction of stromal fibroblast activity [292,297]. Moreover, KRAS-driven metabolic rewiring can dysregulate retinoid-metabolizing enzymes such as ALDH1A and CYP26, leading to RA depletion and cell signaling impairment [294,295]. All these events induce a permissive tissue microenvironment for cancer progression and stromal remodeling.

### 7.2. Modulation of Redox Cell Biology by Retinoic Acid

RA exhibits a crucial role in modulating oxidative stress via both direct and indirect mechanisms [288,289]. This fat-soluble vitamin stimulates transcription of antioxidant enzymes such as superoxide dismutase (SOD) [298], catalase [299], and glutathione peroxidase [300,301] via RAR-mediated expression [294,296]. It can also downregulate NADPH oxidase function, thereby reducing ROS generation from non-mitochondrial origins [302,303,304]. Furthermore, RA interacts with NRF2 signaling [305]: it can enhance NRF2 transcription under physiological conditions, but paradoxically, it normalizes aberrant NRF2 activation in malignant cells, restoring redox balance without fostering their survival [306,307]. In PDAC, *KRAS* and mutated *TP53* stimulate persistent ROS generation and induce cell stress [9,22,23,24]. RA can attenuate oncogenic ROS signaling and, in turn, sensitize cells to oxidative injury and apoptosis [294,295,296,308]. This fat-soluble vitamin regulates mitochondrial biogenesis and antioxidant capacity [309]. Therefore, it influences the mitochondrial setpoint of cellular redox balance [310,311] and shifts cancer cells away from a state of elevated tolerance to ROS that promotes the development of aggressive phenotypes [23,311,312]. Overall, these events have a profound impact on malignant cell survival and on the response to ROS-dependent treatments, such as radiotherapy and certain chemotherapeutic drugs [313,314].

### 7.3. Effects on Stromal Remodeling and CAF Biology

One of the most intriguing roles of RA in PDAC hinges on its ability to rewire tumor-associated fibroblasts (CAFs) [315,316]. These cells are crucial contributors to desmoplasia, hypoxia, and treatment resistance to [7,35,281,282,283]. RA may promote a quiescent status in pancreatic stellate cells and activated CAFs [317]. The features of this phenotype include a reduced production of α-smooth muscle actin (α-SMA) and ECM, as well as the normalization of oxygen diffusion in pancreatic cancer tissue [318]. This “stromal reprogramming” is associated with the restoration of tissue perfusion, the reduction in hypoxia, and the modification of metabolic interplay between cancer cells and the stroma [291,319].

**Figure 5:**
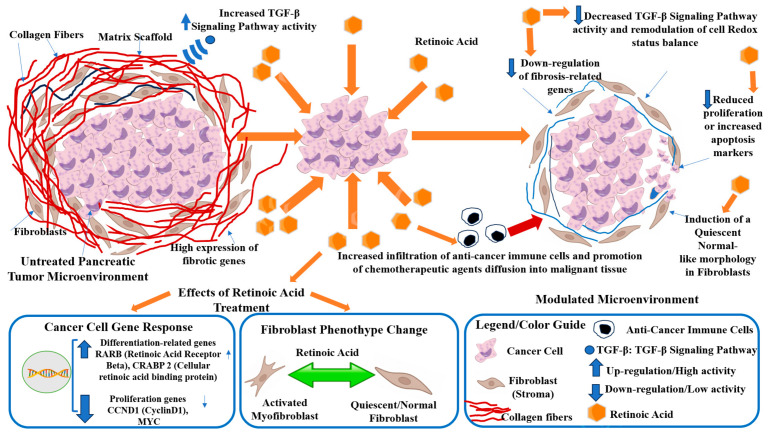
Potential role of RA in counteracting pancreatic carcinogenesis. This fat-soluble vitamin modulates cellular oxidative stress, as it reduces ROS generation through several mechanisms: (1) by increasing the transcription of antioxidant enzymes, such as superoxide dismutase (SOD), catalase, and glutathione peroxidase via RA-mediated expression; (2) by downregulating the activity of NADPH oxidase; (3) by influencing NRF2 signaling, as it can enhance NRF2 transcription under physiological conditions, it normalizes dysregulated NRF2 activation in cancer cells, restoring redox balance without hampering their survival. In PDAC cells, mKRAS and mutated p53 promote persistent ROS production and induce cell stress. RA downregulates oncogenic ROS signaling, sensitizing cells to oxidative damage and apoptosis, influences the mitochondrial setpoint of cellular redox balance [283,284], and shifts cancer cells away from the state of elevated tolerance to ROS associated with more aggressive phenotypes. In PDAC tissue, RA reprograms tumor-associated fibroblasts (CAFs), which are crucial components of desmoplasia, hypoxia, and treatment resistance. RA may induce a quiescent status in pancreatic stellate cells and activated CAFs, promoting: (1) decreased synthesis of α-smooth muscle actin (α-SMA) and ECM; (2) normalization of oxygen diffusion in pancreatic cancer tissue; (3) downregulation of some pro-fibrotic signaling pathways, such as TGF-β and Hedgehog cascades; (4) reduction in CAF-mediated release of cytokines, such as IL-6 and CXCL12. This “stromal rewiring” may improve tissue perfusion, thereby decreasing hypoxia and remodulating metabolic interplay between cancer cells and the stroma. Overall, these events attenuate two crucial steps in carcinogenesis: the pro-tumorigenic inflammation and the immune exclusion. Therefore, RA rewires the malignant microenvironment, making it more permissive to the diffusion of anticancer drugs and to the infiltration of immune cells into neoplastic tissue. RA can synergize with therapies that stimulate ROS production, reversing the Warburg effect and reducing lactate transport from CAFs to malignant cells. These events modulate mitochondrial metabolism and can alter the redox-adapted state of KRAS-mutated malignant cells. In combination with PRMT5 inhibitors or p53-reactivating drugs, RA may increase PUMA-mediated apoptosis. Vitamin A affects mitochondrial activity and reactivates a stromal quiescent phenotype in malignant tissue via modulation of NRF2, thereby sensitizing KRAS-mutant cells to oxidative stress and p53-mediated cancer cell death. RA-mediated modulation of redox balance can promote PUMA activation, thereby inducing mitochondrial outer membrane permeabilization and apoptosis in resistant malignant cells. Furthermore, RA downregulates PRMT5 expression in certain contexts by reducing histone protein methylation and by activating genes involved in metabolic processes. Through this multifaceted interplay, RA disrupts the integrated metabolic–apoptotic–stromal network that supports the aggressiveness of pancreatic ductal carcinoma. This fat-soluble vitamin acts as a therapeutic modulator rather than a direct cytotoxic agent.

Overall, RA acts via the RARβ pathway in CAFs, decreasing pro-fibrotic signaling, such as TGF-β and Hedgehog cascades [236,285,320]. It also downregulates CAF-mediated release of cytokines, including IL-6 and CXCL12 [320,321]. These processes attenuate two crucial events in carcinogenesis: the pro-tumorigenic inflammation and the immune exclusion [322,323]. Therefore, RA does not merely function as a cytotoxic drug, but it reprograms the malignant microenvironment, making it more permissive to the diffusion and infiltration of anticancer agents and to the infiltration of immune cells into neoplastic tissue [22,265,324,325] (Figure 5).

In particular, Retinoic Acid orchestrates proper immune function across both its innate and adaptive arms. In particular, dendritic cells and macrophages are the major sources of retinoic acid for the various subsets of B and T cells. During the early steps of the immune response, these phagocytes interact with naive T cells in the presence of a wide range of biochemical and physical stimuli in the microenvironment, such as cytokines, ROS, costimulatory molecules, retinoic acid, as well as tissue stiffness, viscosity, and shear stress. This complex interplay orchestrates differentiation of T lymphocytes into distinct subclasses, each with different specificities of action. According to the current evidence the effects mediated by this micronutrient are dose-dependent, in permissive contexts, low levels of retinoic acid stimulate T cells to differentiate towards a Th17 phenotype, whereas higher amounts of this compound induce the generation of Th2 and T regulatory cells, therefore preventing strong and potentially dangerous immune responses for the host, Therefore, retinoic acid may contribute to overcome the Cancer Associated Fibroblasts-mediated exhaustion of specific T subsets, rebalancing immune cells function and promoting restoration of immune system homeostasis. These processes have been described in patients suffering from both infectious diseases and malignancies. Strategies combining RA with antineoplastic drugs may make tumor cells more sensitive to treatment and improve progression-free survival in patients [326,327,328,329,330].

### 7.4. Therapeutic Potential in Preclinical PDAC Models

In preclinical PDAC studies, RA use has been shown to be associated with promising results in a wide series of contexts [37,38,331]. RA administration in human and animal cell cultures has been shown to modulate the expression of over 300 miRNAs, thereby reducing tumor cell invasion and inhibiting tumor growth [332,333]. In murine models, stromal remodeling may improve intratumoral oxygenation by promoting the entry of chemotherapeutic drugs such as gemcitabine into tumor tissue [334,335]. Furthermore, RA can synergize with therapies that stimulate ROS generation [335,336]. These treatments exploit the cancer vulnerability originating from the shift in redox balance [137,337].

Moreover, RA modulates the metabolic interplay existing between cancer and stroma, reversing the Warburg effect and decreasing lactate transport from CAFs to malignant cells [246,320]. These events modulate mitochondrial metabolism [31,311] and can alter the redox-adapted state of KRAS-mutated malignant cells [8,103,327]. In combination with PRMT5 inhibitors or p53-reactivating drugs, RA may further enhance PUMA-mediated apoptosis, underscoring the cross-talk between metabolic and apoptotic networks [338,339,340].

### 7.5. Clinical Trials of Retinoic Acid and Retinoids in PDAC

Despite very interesting reports on the possible anticancer activity of RA, clinical trials assessing its anticancer activity in PDAC remain limited, but some studies suggest a potentially interesting application in the management of this malignancy [341]. However, to date, the results of both in vitro and in vivo studies remain preliminary, and further research is needed to either confirm them or refute them. Early phase studies investigated all-trans retinoic acid (ATRA) as a therapy to induce cell differentiation in advanced PDAC, with modest activity and good tolerability [292,342]. Recent studies have focused on combination strategies. For example, ATRA administration in combination with gemcitabine and nab-paclitaxel has been assessed in phase I/II settings [156,334,335,338] and has shown improved stromal restoration and increased drug penetration compared with chemotherapy alone [343,344]. Biomarker analyses from trials suggest that ATRA may increase RARβ expression and promote stromal quiescence in patients who respond to this treatment [317,318]. Furthermore, synthetic agents and RAR agonists with improved pharmacokinetic properties and increased selectivity, as well as nanoparticle delivery systems, are being investigated in clinical trials to overcome the limitations in the anticancer efficacy of currently available drugs [345,346,347].

Although the clinical efficacy of RA remains under investigation, these strategies underscore the translational potential of RA-based stromal targeting in PDAC [39,341].

### 7.6. Cross-Talk Among KRAS, p53, PUMA, PRMT5, Retinoic Acid and Mitochondrial Activity

Vitamin A affects mitochondrial activity and reactivates a stromal quiescent phenotype in malignant tissue [315,317,348,349], via modulation of NRF2 [28,143,145]. In particular, this fat-soluble vitamin indirectly sensitizes KRAS-mutant cells to oxidative stress and p53-mediated cancer cell deaths [349,350]. RA-mediated modulation of redox balance can promote PUMA activation, thereby inducing mitochondrial outer membrane permeabilization and apoptosis in resistant malignant cells [351,352]. Furthermore, RA has been shown to downregulate PRMT5 expression in certain contexts by reducing histone protein methylation, which mediates the activation of metabolic genes [338,339,340]. Through this multifaceted crosstalk, RA disrupts the integrated metabolic–apoptotic–stromal network that supports the aggressiveness of pancreatic ductal carcinoma, making it plausible that this fat-soluble vitamin acts as a therapeutic modulator rather than a direct cytotoxic agent [103,327,338,339,340].

## 8. Discussion

PDAC remains one of the most severe and therapy-resistant cancers, driven by a complex cross-talk among a wide series of factors, including oncogenic mutations, metabolic rewiring, redox adaptation, and malignant microenvironment remodeling therapies [176,190,291,320]. The interplay developing among *KRAS* and *TP53* mutations, mitochondria, hypoxia, ROS, and stroma orchestrates tumor onset, progression, spreading, and response to therapies [349,353]. A proper understanding of these interconnected networks is critical to uncovering points of cancer susceptibility and designing adequate and effective treatments against PDAC [349,353]. 

*KRAS* mutations are common in PDAC and induce constitutive activation of the MAPK and PI3K pathways, promoting cell proliferation and metabolic restoration [69,70,71,157]. Furthermore, *TP53* mutations, detectable in 50–75% of patients with pancreatic cancer, alter a crucial apoptotic checkpoint and promote adaptation of malignant cells to metabolic and oxidative stress [1,6,7,15,16,17,18,19]. Overall, these changes cause a shift toward aerobic glycolysis, known as the Warburg effect, and also maintain mitochondrial function, promoting the anabolic function and redox regulation of malignant cells [20,21,22,23,24,25]. KRAS-mediated metabolic cell remodulation increases glucose uptake, the glycolytic rate, and the diversion of glutamine metabolism via *anaplerosis*, thereby generating ATP and precursor molecules [18,19,21,40,68,353]. Furthermore, mitochondrial function is preserved to meet anabolic needs and produce ROS, which act as second messengers to promote tumor development [82,84,85,86,117,301,354]. Mutant p53 further enhances mitochondrial fitness and antioxidant activity, enabling cells to survive in oxidative and nutrient-deprived environments [351,353,354]. Overall, these events promote a pro-oxidant, highly buffered state that suppresses apoptosis and promotes the progression of malignancy [26,27,355].

The microenvironment in PDAC consists of a poorly perfused, fibrous stroma with extreme heterogeneity in oxygen distribution within the malignant tissue [281,356]. Hypoxic regions stabilize the HIF transcription factor, increasing glycolysis and promoting an aggressive biological behavior [85,115,116,118,280]. Cancer-associated fibroblasts metabolically support malignant cells via the production and release of lactate and alanine. These substrates act as energy suppliers for tumor cells [73,257,258,260]. Hypoxia also suppresses immune system activity via adenosine signaling and alters T cell function [7,73,98,334,354,355,356]. These events further promote PDAC resistance to the host’s anticancer mechanisms and to specific anti-neoplastic treatment [70,79,130]. Oxygen gradients between the malignant epithelium and the stroma strongly influence redox metabolism [79,82,85,118,356]. PDAC cells located in moderately oxygenated regions can still support mitochondrial activity and ROS signaling. On the other hand, the metabolism of tumor cells residing in severely hypoxic regions strongly depends on glycolysis and stromal support [6,349,357].

A crucial link between metabolism and apoptosis is modulated by PUMA, a BH3-only proapoptotic protein. Its activity is regulated by p53 and oxidative stress [29,31,32,35,36]. Under normal conditions, PUMA functions as a redox checkpoint, triggering the early steps of mitochondrial apoptosis in cells undergoing to excessive oxidative stress [32,33,34,35]. However, in PDAC, this checkpoint is frequently suppressed by *TP53* mutations, PUMA silencing, and excessive activation of the antioxidant network [29,31,32]. PRMT5, an epigenetic modulator, further increases cancer resistance by suppressing proapoptotic genes and activating antioxidant cascades [30,70,179,197,198,199,200,308]. This enzyme promotes the survival of KRAS-mutated cells by regulating mitochondrial metabolism and redox buffering capacity [352,358]. The combination of two events, including suppression stress of PUMA [304] and activation stress of PRMT5 [204,218], allows malignant pancreatic cancer cells to survive in hostile, potentially lethal tissues and to develop treatment-resistant cellular phenotypes [85,112,120,199].

Taking into account all the considerations reported in this article, multiple possible antitumor therapeutic options and strategies can be developed. An appropriate strategy is to target mitochondrial metabolism or antioxidant defense systems to selectively kill redox-dependent KRAS-mutated malignant pancreatic cells [353,354,355,359]. Drugs with PRMT5 inhibitory activity can disrupt epigenetic and metabolic support networks [179,197,198,199,200], promoting ROS generation and restoring apoptotic cascades [33,201,202,203,216]. p53 recovery strategies (e.g., APR-246) can restore PUMA-induced and mitochondrial-mediated apoptosis [360]. Retinoic acid (RA) plays a unique role as a non-cytotoxic regulator of redox and matrix biology [289,290]. RA may increase the sensitivity of malignant cells to chemotherapy and radiotherapy by promoting a quiescent phenotype in cancer-associated fibroblasts (CAFs) [291,317,318], normalizing oxygen delivery, and reprogramming antioxidant signaling cascades [287,290,291,294,297,302,303,304]. A therapeutic strategy combining RA, ROS-generating drugs, PRMT5 inhibitors, or metabolic treatments can induce synergistic effects by simultaneously altering cancer cell metabolism and restoring sensitivity to apoptotic mechanisms [290,291]. Furthermore, strategies aimed at modulating oxygen tension delivery in neoplastic pancreatic tissue [361], including stromal depletion [193,332], vascular normalization, or the use of hypoxia-activated compounds, may overcome intrinsic resistance to anti-cancer treatment by modifying the characteristics of the redox environment [287,290,291,294,297].

Despite these promising findings, several challenges persist. Three points have been underlined: (1) the metabolic plasticity of PDAC ensures a rapid adaptation to antineoplastic therapeutic action, requiring multidisciplinary strategies [70,308]; (2) heterogeneity in pancreatic cancer tissue oxygenation and stromal composition across patients and tumors prevents the use of common anticancer strategies [199,359]; (3) drug entry in malignant lesions remains limited by desmoplasia, although stromal reprogramming with RA and similar drugs may provide useful effects [36,37,308,346,347].

However, despite the aforementioned therapeutic benefits, some studies have reported that Vitamin A and retinoic acid may exhibit non-univocal effects, including acute metabolic dysregulation and a delayed cancer-promoting effect [362]. Some cohort studies and meta-analyses have suggested that both excessive and insufficient vitamin A intake may increase cancer risk in various malignancies. Therefore, further in vitro and in vivo studies are needed to improve our understanding of the potential role of this micronutrient, in combination with chemotherapy, for the treatment of human malignancies in general and for the management of pancreatic cancer in particular.

## 9. Conclusions

PDAC provides an example of the convergence of oncogenic signaling, metabolic adaptation, cellular redox status modulation, and stromal remodeling in driving malignancy initiation and spread, as well as the development of therapeutic resistance in neoplasms. Central elements in this network are represented by mutated KRAS and p53, which, in concert, reprogram glucose and glutamine metabolism, support mitochondrial activity under stress, and reshape the cellular redox balance. This metabolic rewiring promotes aggressive cancer behavior in the presence of a hypoxic microenvironment, nutrient reduction, and dense desmoplasia, characteristics that would normally induce apoptosis in non-neoplastic cells.

Some regulatory molecules, such as PUMA and PRMT5, function as molecular switchers linking redox stress and apoptosis. Meanwhile, oxygen gradients within the malignant tissue and CAF-mediated metabolic supply generate spatial recesses with distinct vulnerabilities, emphasizing the need for therapies that target intra-neoplastic heterogeneity.

Future trials should focus on the use of integrated treatment approaches with the aim of defining metabolic, redox, epigenetic, and stromal pathways. The possible therapeutic options should consider combinations of the following measures: PRMT5 inhibition + RA, p53 reactivation + ROS-inducing chemotherapy, and stromal normalization + immunotherapy. Well-designed precision trials, including patients with PDACs with properly profiled metabolic, genetic, and stromal characteristics, may enable stratification of patients who will likely benefit from these interventions and identify a useful and effective dosage of vitamin A for this type of malignancy. Clinical trials should be designed to include assessment of redox status, oxygenation, and stromal activity to predict response to therapy and patient outcomes.

## Data Availability

No new data were created or analyzed in this study.

## Abbreviations

The following abbreviations are used in this manuscript:

AKT	Protein kinase B
ATP	Adenosine Triphosphate
BAK	Bcl-2 homologous antagonist/killer
BAX	Bcl-2-associated X protein
BCL-2	B-cell lymphoma 2
CAF	Cancer-associated Fibroblast
CDKN2A	Cyclin-dependent kinase inhibitor 2A,
ERK	Extracellular signal-regulated kinase
GCLC	Glutamate–cysteine ligase catalytic subunit
GDP	Guanosine Diphosphate
GTP	Guanosine Triphosphate
HIF-1α	Hypoxia-inducible factor 1-alpha
HO-1	Heme oxygenase 1
KEAP1	Kelch-Like ECH-Associated Protein 1
KRAS	Kirsten rat sarcoma viral oncogene homolog
mTOR	Mammalian Target of Rapamycin
MEK	Mitogen-activated protein kinase kinase
NQ01	Nicotinamide adenine dinucleotide (phosphate) reduced:quinone oxidoreductase
Nrf2	Nuclear factor erythroid 2-related factor 2
p53	Protein p53
PDAC	Pancreatic ductal adenocarcinoma
PI3K	Phosphoinositide 3-kinase
PRMT5	Protein Arginine Methyltransferase 5
PUMA	p53-upregulated modulator of apoptosis
RA	Retinoic Acid
RAF	Rapidly Accelerated Fibrosarcoma
ROS	Reactive oxygen species
RXR	Retinoid X Receptors
SMAD4	Mothers against decapentaplegic homolog 4
TAM	Tumor-associated macrophages
TME	Tumor microenvironment
TIGAR	TP53-Induced Glycolysis and Apoptosis Regulator
TP53	Tumor Protein 53
